# TGFBI in tumors and the tumor immune microenvironment: functional roles, mechanisms, and therapeutic targeting

**DOI:** 10.3389/fimmu.2026.1880488

**Published:** 2026-07-23

**Authors:** Jingwen Li, Yuecheng Zeng, Feng Chen, Jinyang Hu, Zhongyin Guo, Peng Peng

**Affiliations:** 1 Department of Oncology, Xiangyang Central Hospital, Affiliated Hospital of Hubei University of Arts and Science, Xiangyang, China; 2 Department of Neurosurgery, Xiangyang Central Hospital, Affiliated Hospital of Hubei University of Arts and Science, Xiangyang, China; 3 Wenzhou Medical University, Wenzhou, China; 4 Department of Neurosurgery, The Second Affiliated Hospital of Wenzhou Medical University, Wenzhou, China

**Keywords:** extracellular matrix, TGFBI, therapeutic target, tumor immune microenvironment, tumorigenesis

## Abstract

TGFBI in tumors, providing a comprehensive theoretical foundation for developing TGFBI-based precision diagnosis and treatment. Transforming growth factor-beta-induced protein (TGFBI) is a secreted extracellular matrix protein involved in various physiological and pathological processes. Its multiple roles in tumorigenesis and the remodeling of the tumor immune microenvironment (TIME) have recently drawn increasing attention. This review systematically compares the dual pro-tumor and tumor-suppressive functions of TGFBI across multiple cancer types and provides a comprehensive analysis of its immunomodulatory roles—particularly its recently identified function as a secreted immune checkpoint bridging extracellular matrix remodeling and immune suppression. By integrating evidence from single-cell and spatial transcriptomic studies, we elucidate the cell-type-specific expression patterns of TGFBI in the tumor microenvironment and propose a tumor-type-specific therapeutic stratification framework for TGFBI-targeted interventions. This review thus fills a critical gap by providing the first systematic integration of TGFBI’s context-dependent functions with its translational implications.

## 1 Introduction

TGFBI (also known as βig-h3) was first identified in 1992 as a transforming growth factor-β (TGF-β)-responsive gene in A549 lung adenocarcinoma cells, and the encoded protein was subsequently characterized as a secreted extracellular matrix (ECM) protein with four FAS1 domains and an RGD motif (1, 2). As a TGF-β-induced ECM protein, TGFBI is an important regulator of cellular physiological functions such as adhesion and migration. In tumor biology, TGFBI has complex dual properties: its function depends on the microenvironment, but in the malignant stage of tumors, it mainly manifests as a pro-tumor effect. Clinical observations have found that TGFBI is significantly upregulated in various cancers, including colorectal cancer, pancreatic cancer, and breast cancer, and has become a key indicator for assessing tumor progression and prognosis (3–5).This protein not only directly supports tumor cell proliferation and anti-apoptosis but also, by promoting angiogenesis, serves as a core driving force for cancer invasion and metastasis (6).

Beyond its direct effects on tumor cells, TGFBI is also a core molecule in the construction and regulation of the tumor microenvironment (TME). This regulatory effect largely mediates the interaction between tumor cells and stromal cells, especially tumor-associated macrophages, (TAMs). In ovarian cancer progression, TAMs highly express TGFBI in both the periphery of early lesions and late-stage tissues, thereby synergistically driving tumor progression by remodeling the immunosuppressive microenvironment (7). Single-cell sequencing further confirmed that in pancreatic cancer, TGFBI is a key secreted factor through which TAMs exert their pro-tumor effects: knockout of this protein effectively blocks macrophage polarization toward the M2 type, thereby enhancing antitumor immune responses and increasing chemotherapy sensitivity (8). Furthermore, in glioblastoma, TGFBI acts as a communication link maintaining the tumor niche: it mediates the paracrine support of stem cell stemness by M2-type TAMs (9),and can also participate in an autocrine positive feedback loop in cancer stem cells under hypoxic conditions, thereby consolidating the tumorigenic capacity of the tumor in multiple dimensions (10).

The immunomodulatory effects of TGFBI further extend to the adaptive immune system. In clear cell renal cell carcinoma, tumor cell-secreted TGFBI recruits regulatory T cells (Tregs) by inducing the expression of chemokine CCL22, mediating the local immunosuppressive process (11). A more groundbreaking study has found that TGFBI possesses the characteristics of a novel secreted immune checkpoint: it is widely expressed in various lymphocytes and restricts T cell activation, functional differentiation, and tissue homing through an autocrine inhibitory loop; neutralizing TGFBI significantly reverses this inhibition (12). This broad immunomodulatory capacity has also been confirmed in non-tumor fields (13). In addition to transmitting biochemical signals, TGFBI also forms an immune barrier by shaping the physical properties of the extracellular matrix (ECM). In liver fibrosis, TGFBI mediates a positive feedback loop between stellate cells and macrophages, activating the PDGFRβ (platelet-derived growth factor receptor beta) pathways via integrin αvβ3, increasing matrix stiffness, and maintaining a pro-fibrotic environment (14). In colorectal cancer, this matrix remodeling participates in forming the typical “immune exclusion” phenotype, where its physical barrier and biochemical effects together limit the infiltration of cytotoxic immune cells, and this process is highly synchronized with the progression of chromosomal instability (15).

Given its central role in tumor progression, stemness maintenance, immunosuppression, and matrix remodeling, TGFBI has emerged as a novel therapeutic target with high potential for clinical translation. Preclinical studies have preliminarily validated the feasibility of targeting this molecule: anti-TGFBI antibody therapy effectively reduces tumor burden in high-grade serous ovarian cancer and reshapes the immune microenvironment by activating intratumoral β3-positive unconventional T cells (7).In bladder cancer, blocking the hypoxia-induced TGFBI/GDF15 (growth differentiation factor 15) axis effectively inhibits the self-renewal of tumor stem cells and significantly enhances the efficacy of chemotherapy (16). Furthermore, due to the complex interplay between pathways such as endogenous PD-L1 (programmed death-ligand 1) and TGFBI, TGFBI also holds promise as a biomarker for assessing tumor progression and guiding precision therapy (17). In summary, intervention strategies targeting TGFBI are expected to reshape the tumor niche, synergistically enhance the effects of immune checkpoint inhibitors and chemotherapeutic agents, and provide a new breakthrough in overcoming tumor resistance.

## 2 Molecular structure and expression regulation of TGFBI

### 2.1 Gene and protein structural characteristics of TGFBI

The TGFBI gene, located at 5q31, encodes a key secreted extracellular matrix protein (18).In terms of domain composition, the protein consists of an N-terminal EMI domain (named after the EMILIN proteins, characterized by an N-terminal cysteine-rich domain), four tandem FAS1 domains, and a C-terminal RGD motif, which are responsible for protein-protein interactions, receptor-mediated signal transduction, and integrin recognition, respectively (19).Studies on the fission yeast homologous protein Fsc1 and structural predictions indicate that the FAS1 domain serves as an extended scaffold, and the integrity of its structure is critical for maintaining protein function (19, 20).More than 70 TGFBI mutations have been clinically reported to date, mainly distributed in the FAS1 domain. These mutations (particularly p.Arg124Cys and p.Arg555Trp) induce misfolding by disrupting the thermodynamic stability of the protein and hinder proteolytic clearance, leading to its abnormal deposition in the cornea as amyloid or amorphous material (18, 21–23).This domain-based complex interaction enables TGFBI to exert multidimensional regulatory roles in maintaining corneal transparency, pathological angiogenesis, and tumor progression (24–26).

### 2.2 Differential expression and clinical significance of TGFBI in various tumor types

TGFBI is highly expressed in various malignant tumors, and its levels are significantly correlated with tumor aggressiveness and poor prognosis. In ovarian cancer, high TGFBI expression predicts shortened overall survival (OS), progression-free survival (PFS), and post-progression survival (PPS); it mediates tumor metastasis by regulating EMT markers and ECM-degrading proteins, and is recognized as an independent prognostic factor (27).In nervous system and digestive system tumors, TGFBI also exhibits key pro-tumor effects: in glioblastoma multiforme, its expression is closely associated with ECM assembly, angiogenesis, and dendritic cell infiltration (28);In colorectal cancer, TGFBI acts as a core hub gene involved in tumor progression and the EMT process, with significant diagnostic and prognostic value (29, 30).Furthermore, the pro-tumor potential of TGFBI has also been validated in urological, respiratory, and head and neck tumors: in clear cell renal cell carcinoma, it is incorporated as an immune-related prognostic factor into a urine-based prognostic model (4, 31, 32);In breast cancer, head and neck squamous cell carcinoma, and oropharyngeal squamous cell carcinoma, overexpression of TGFBI is often accompanied by lymph node or distant metastasis and is a key biomarker for assessing survival benefit (33–37). In summary, in most epithelial-derived tumors, TGFBI is a core molecule reflecting tumor invasiveness and progression status.

However, the biological functions of TGFBI exhibit significant tissue specificity and can act as a tumor suppressor in certain contexts. Its expression is tightly regulated by epigenetic mechanisms, particularly DNA methylation in the promoter region. In Burkitt lymphoma, Epstein-Barr virus combined with aflatoxin B1 induces methylation-mediated silencing of the TGFBI gene by recruiting DNMT1, thereby driving malignant transformation of B cells (38, 39). Bioinformatic analyses and patient cohort studies suggest that this loss of expression is also observed in non-small cell lung cancer and some gastric cancers, and low expression of TGFBI is also associated with poor prognosis, suggesting that it exerts a potential growth-inhibitory function in these types of tumors (39).This difference in expression patterns indicates that the function of TGFBI is highly dependent on the intracellular signaling pathway context, and clinical interpretation of its expression levels must take into account the specific tumor type.

Overall, the expression patterns and functional roles of TGFBI exhibit complex context dependence. In cancer types with clear pro-tumor effects such as ovarian cancer, glioblastoma, and colorectal cancer, high expression of TGFBI can effectively assess shortened survival and increased invasiveness, making it suitable for integration into multi-gene prognostic models or clinical prediction feature sets (4, 27, 28, 30).For example, in pancreatic cancer, it mediates EMT by activating the PI3K/AKT pathway and is associated with perineural invasion (40); in the tumor immune microenvironment, TGFBI can even act as a secreted immune checkpoint, inducing immunosuppression by recruiting Tregs (11, 12).Conversely, in tumors represented by Burkitt lymphoma, loss of its function (such as methylation silencing) is the driver of the malignant phenotype. Therefore, the value of TGFBI as a biomarker is not universal, and its clinical application must be based on a deep understanding of the specific tumor biological context. Its expression can reflect either pro-tumor ECM remodeling and immune evasion or the loss of tumor suppressor function, and this multidimensional role makes it a therapeutic target in need of further exploration in precision medicine. (**Table 1**).

**Table 1:** Tumor-type-specific expression, mechanisms, and functions of TGFBI.

Cancer type	TGFBI expression	Major cellular source	Receptor/integrin Involved	Downstream pathway	Immune cell effect	Clinical association	Functional classification
Ovarian Cancer	Upregulated	TAMs, tumor cells	αvβ3	PI3K/Akt, FAK	M2 polarization, NK suppression, Treg recruitment	Poor OS, PFS, PPS; chemotherapy resistance	Pro-tumor
Colorectal Cancer	Upregulated	Tumor cells, stromal cells	αvβ3/αvβ5	FAK/PI3K/Akt, MAPK/ERK	Immune exclusion, CTL barrier	Diagnostic and prognostic marker	Pro-tumor
Glioblastoma	Upregulated	M2 TAMs, cancer stem cells	αvβ3	PI3K/Akt	M2 polarization, stemness maintenance	Poor prognosis	Pro-tumor
Pancreatic Cancer	Upregulated	TAMs, CAFs	αvβ3	PI3K/AKT, MAPK/ERK	M2 polarization, immunotherapy resistance	Perineural invasion, poor prognosis	Pro-tumor
Clear Cell RCC	Upregulated	Tumor epithelial cells	Unknown	CCL22 upregulation	Treg recruitment	Prognostic factor	Pro-tumor
Breast Cancer	Upregulated	Tumor cells	αvβ3/αvβ5	FAK/PI3K/Akt	Unknown	Lymph node/distant metastasis	Pro-tumor
HNSCC	Upregulated	Tumor cells (p-EMT)	Unknown	EMT (Snail, Twist)	Unknown	Lymph node metastasis	Pro-tumor
Lung Adenocarcinoma	Context-dependent (PD-L1 axis)	Tumor cells	Unknown	PD-L1-TGFBI-CITED2-p21	Unknown	Growth arrest	Tumor-suppressive

## 3 The dual functions and mechanisms of TGFBI in tumorigenesis and development

### 3.1 Pro-tumor mechanisms of TGFBI

**Figure 1:**
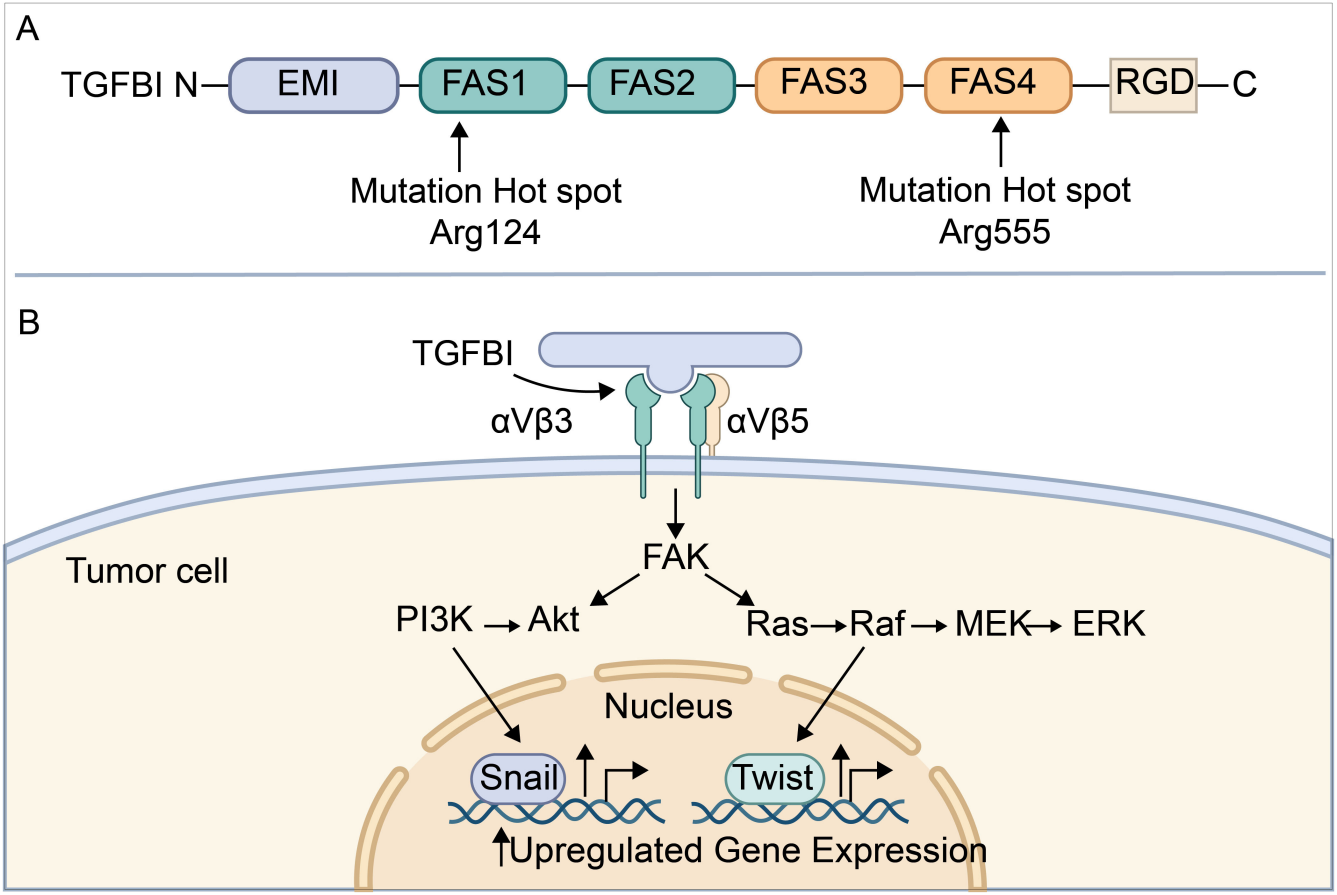
Molecular structure of TGFBI and integrin-mediated intracellular signaling pathways. **(A)** Domain organization of TGFBI. The protein consists of an N-terminal EMI domain (named after the EMILIN proteins, characterized by a cysteine-rich region), four tandem FAS1 domains, and a C-terminal RGD motif, which are responsible for protein-protein interactions, receptor-mediated signal transduction, and integrin recognition, respectively. Clinically reported mutations (Arg124Cys, Arg555Trp) are predominantly located within the FAS1 domains and are associated with corneal dystrophies. **(B)** TGFBI-integrin interaction and downstream signaling. Secreted TGFBI binds to specific integrin receptors (αvβ3, αvβ5, α3β1) on the tumor cell surface, activating FAK/PI3K/Akt and MAPK/ERK signaling cascades. These pathways upregulate transcription factors such as Snail and Twist, driving epithelial-mesenchymal transition (EMT), enhancing cell proliferation, and conferring resistance to apoptosis.

TGFBI exerts its pro-tumor functions primarily through integrin-mediated signaling, EMT promotion, angiogenesis, and matrix stiffness regulation. TGFBI specifically binds to integrins αvβ3, αvβ5, and α3β1, which are highly expressed on the surface of tumor cells, and activates downstream pro-survival and pro-proliferation signaling axes such as FAK/PI3K/Akt and MAPK/ERK (14). In renal cell carcinoma, TGFBI significantly enhances the proliferative viability of tumor cells through the PI3K/AKT/mTOR/HIF-1α pathway (40). In hepatocellular carcinoma, the TGFBI/MAPK/ERK axis mediated by the oncogene EIF3B is a key link in accelerating cell invasion and metastasis (41). This integrin-mediated signaling cascade not only reinforces the growth advantage of tumors but is also closely associated with clinical chemotherapy resistance (42). Furthermore, cancer-associated fibroblasts (CAFs) in the tumor microenvironment can also secrete TGFBI, forming a paracrine positive feedback loop with tumor cells. For example, in pancreatic cancer, CAF-derived TGFBI is regarded as a core effector molecule driving immunotherapy resistance and poor prognosis (43), highlighting the central role of TGFBI-integrin signaling activation in malignant progression. (**Figure 1**).

Meanwhile, TGFBI is also a key driver of epithelial-mesenchymal transition (EMT), enhancing tumor invasion and metastatic potential by inducing phenotypic remodeling. At the molecular level, TGFBI promotes the acquisition of mesenchymal traits in tumor cells by upregulating key transcription factors such as Snail and Twist and suppressing the expression of the epithelial marker E-cadherin (1).Clinical studies have shown that TGFBI is an important marker of partial epithelial-mesenchymal transition (p-EMT) in head and neck squamous cell carcinoma, and its expression level is negatively correlated with lymph node metastasis (35, 36); In renal cell carcinoma, knockdown of TGFBI can significantly reverse the EMT process and inhibit cell migration (40). Notably, TGFBI-induced EMT often exhibits crosstalk with the classic TGF-β signaling pathway: in diffuse large B-cell lymphoma, TGFBI reduces cellular sensitivity to cisplatin by regulating the TGF-β pathway (44); In cholangiocarcinoma, the RNA-binding protein LIN28B activates the TGF-β axis via TGFBI, synergistically enhancing cancer stem cell properties and migratory capacity (45), forming a positive feedback network that synergistically reinforces EMT. Furthermore, TGFBI plays an important role in regulating matrix stiffness and metastatic dissemination. In liver fibrosis and various solid tumors, TGFBI secreted by activated stellate cells and tumor cells binds to integrin αvβ3 on macrophages and fibroblasts, activating downstream FAK and ERK signaling to promote collagen deposition and ECM crosslinking, thereby increasing tissue stiffness. This mechanical remodeling enhances focal adhesion assembly in tumor cells and facilitates integrin-dependent migration, creating a positive feedback loop in which TGFBI-driven matrix stiffening promotes further invasion and metastatic dissemination. In the tumor stromal microenvironment, TGFBI also regulates endothelial cell behavior and the release of angiogenic factors through multiple pathways, thereby providing hemodynamic support for tumor growth. TGFBI directly promotes endothelial cell migration and tube formation, Its significantly high expression during the proliferative phase of infantile hemangioma validates its potent pro-angiogenic activity (46). Mechanistically, TGFBI has a synergistic regulatory relationship with key factors such as VEGFA; for example, in cervical cancer, TGFBI is included in a hypoxia-related signature and together with VEGF indicates poor prognosis (47, 48). Nevertheless, the role of TGFBI in the microenvironment is complex: in pancreatic neuroendocrine tumors, loss of stromal TGFBI may instead induce macrophage polarization toward the M2 type, indirectly promoting angiogenesis (49). In precancerous lesions such as liver fibrosis, the pro-fibrotic environment constructed with the involvement of TGFBI is often accompanied by abnormal vascular proliferation, driving the progression of tissue remodeling toward malignant transformation (14). In summary, TGFBI lays the physical foundation for continuous tumor expansion and distant dissemination by finely regulating endothelial cell function and angiogenic signaling.

### 3.2 Mechanisms of TGFBI’s tumor-suppressive functions

Under specific conditions, TGFBI can inhibit tumors by inducing cell cycle arrest and promoting apoptosis. In lung adenocarcinoma, TGF-β-induced PD-L1 upregulates TGFBI, which in turn inhibits FAK activation and affects CITED2, ultimately upregulating p21CIP1 to cause cell growth arrest (17).In colorectal cancer, the tumor suppressor gene FOXS1 promotes lysosomal degradation of TGFBI; restoring TGFBI expression reverses FOXS1-mediated growth inhibition of tumor cells, suggesting that TGFBI has tumor suppressor activity (50).In nephroblastoma, lncRNA H19 downregulates TGFBI via miR-675 while upregulating Caspase-8, thereby inhibiting proliferation and promoting apoptosis (51).In colon cancer, miR-766-3p directly targets and downregulates TGFBI, inhibiting malignant phenotype and inducing apoptosis (52).In Burkitt lymphoma and other B-cell lymphomas, Epstein-Barr virus and aflatoxin B1 mediate TGFBI promoter methylation via DNMT1, leading to its silencing and loss of tumor suppressor function, thereby promoting tumorigenesis (38).These mechanisms collectively indicate that TGFBI can mediate G1 phase arrest and initiate the apoptotic cascade.

**Figure 2:**
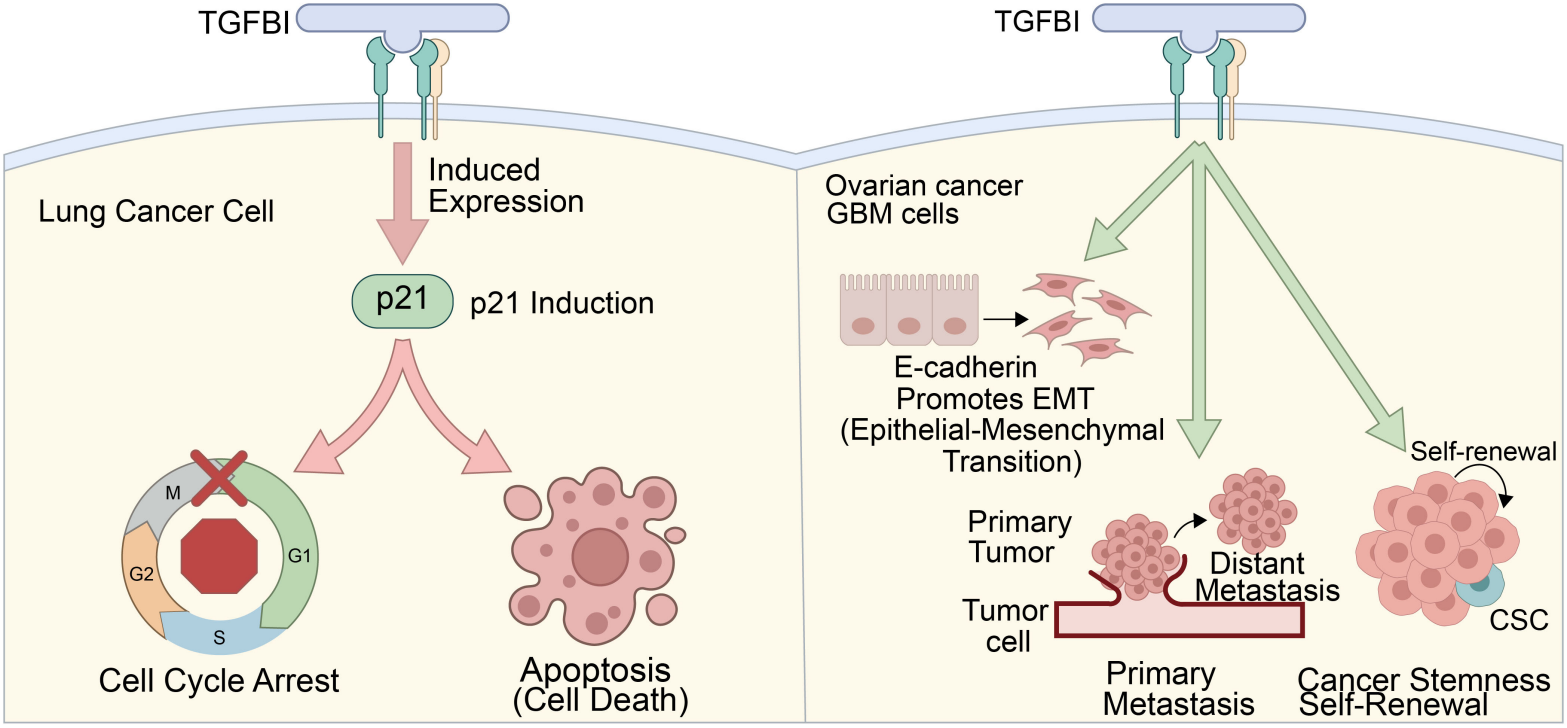
Dual functions of TGFBI in tumorigenesis and its context-dependent roles. TGFBI exhibits significant functional heterogeneity across different tumor types and stages of progression. In certain contexts (such as lung adenocarcinoma or Burkitt lymphoma), TGFBI exerts tumor-suppressive effects by inducing p21CIP1 expression, which mediates cell cycle arrest and initiates apoptotic programs. However, in advanced malignant tumors such as ovarian cancer, glioblastoma, and colorectal cancer, TGFBI predominantly acts as a pro-tumorigenic factor, driving malignant progression by promoting metabolic reprogramming, maintaining cancer stemness, and enhancing cellular invasiveness.

As an extracellular matrix component, high expression of TGFBI can enhance cell adhesion, inhibit anoikis, and form a physical barrier that limits tumor dissemination, an effect that is highly dependent on integrins. Binding of DKK3 to TGFBI inhibits cell migration, indicating that TGFBI limits motility by regulating cell-matrix adhesion (53).In an intervertebral disc degeneration model, TGFBI promotes nucleus pulposus cell apoptosis and ECM degradation, suggesting it affects cell adhesion and survival within the matrix (54).However, in osteosarcoma, tumor cell-derived TGFBI promotes migration and invasion via the ERK pathway, and niclosamide can inhibit this process (55).In renal cell carcinoma, TGFBI promotes proliferation and EMT (40). Thus, the regulation of metastasis by TGFBI is highly context-dependent. The tumor-suppressive role of TGFBI is not universal but depends on cell type, microenvironmental factors, and the integrin subtypes activated. In pancreatic cancer, cancer-associated fibroblast-secreted TGFBI promotes collagen deposition and the formation of a pro-tumor microenvironment (56);Conversely, loss of stromal TGFBI instead promotes M2-type macrophage polarization and angiogenesis, accelerating neuroendocrine tumor progression (49).This indicates that stroma-derived TGFBI is mostly pro-tumor, whereas loss of TGFBI in specific cells can paradoxically be tumorigenic. In ovarian cancer, macrophage-derived TGFBI constructs an immunosuppressive microenvironment via integrin αvβ3 (7).In bladder cancer, hypoxia-induced TGFBI maintains the stemness of cancer stem cells (16).The PD-L1-TGFBI tumor-suppressive axis in lung adenocarcinoma is regulated by TGF-β (17), whereas in diffuse large B-cell lymphoma, TGFBI promotes progression and reduces cisplatin sensitivity through the TGF-β pathway (44). Based on the available evidence from the studies reviewed here, the ultimate function of TGFBI is determined collectively by its expression level, cellular origin, integrin profile, and the tumor microenvironment. This context-dependent duality underscores the necessity of careful patient stratification and tumor-type-specific functional characterization when evaluating TGFBI as a therapeutic target. (**Figure 2**).

## 4 TGFBI shapes and regulates the tumor immune microenvironment

### 4.1 The impact of TGFBI on tumor-associated macrophage polarization

TGFBI plays a key role in inducing the polarization of tumor-associated macrophages (TAMs) toward the pro-tumor M2 type, serving as an important component in constructing the immunosuppressive microenvironment. This process depends on TGFBI binding to integrin receptors on the macrophage surface. In pancreatic cancer, TAMs themselves secrete TGFBI; both *in vitro* siRNA interference and *in vivo* gene knockout significantly inhibit macrophage polarization toward the M2 type (8).In ovarian cancer, tumor cell-derived exosomes carrying thrombospondin 1 (THBS1) promote M2 polarization by regulating TGFBI expression in macrophages (57).TGFBI downstream signaling depends on specific integrins. In glioblastoma, TAM-secreted TGFBI is a key pro-tumor factor in macrophage-tumor cell crosstalk (58).Cathepsin D (Cat D) can accelerate M2 polarization by inhibiting TGFBI, while overexpression of TGFBI attenuates this process, suggesting that TGFBI is a key molecule in regulating macrophage polarization (59).In pancreatic neuroendocrine tumors, loss of TGFBI instead promotes TAM polarization toward M2 and angiogenesis, accelerating tumor progression (49).Single-cell transcriptomic analysis of liver cancer shows that TGFBI is a core gene regulating macrophage polarization and holds potential as a therapeutic target (60).

M2-type macrophages regulated by TGFBI suppress the function of immune effector cells such as cytotoxic T cells by secreting anti-inflammatory factors including IL-10 and TGF-β and highly expressing arginase 1, thereby forming an immunosuppressive microenvironment (8).In ovarian cancer, cancer-associated mesenchymal stem cells (CA-MSCs) recruit and induce macrophages to polarize toward an immunosuppressive phenotype; macrophages with high TGFBI expression directly suppress NK cell activity, leading to immune exclusion and resistance to immune checkpoint inhibitors (61).Single-cell analysis of cervical squamous cell carcinoma indicates that TGFBI-positive TAMs are hypoxia-related monocyte-derived M0-like macrophages and are associated with poor prognosis and activation of tumor proliferation pathways (62).In glioblastoma, an interferon-γ-related gene signature that includes TGFBI can indicate an immunosuppressive “inflammatory phenotype” and is associated with clinical prognosis (63).In ovarian cancer, high TGFBI expression is closely associated with chemotherapy resistance and poor prognosis (42).Due to the central role of TGFBI in driving M2 polarization and immunosuppression, targeting the TGFBI pathway can reprogram the tumor microenvironment and enhance antitumor immunity. Blocking the binding of TGFBI to receptors on macrophages can reverse M2 polarization and restore the antitumor activity of macrophages. In pancreatic cancer, knockout of TGFBI in macrophages inhibits M2 polarization, enhances antitumor immunity, and increases chemotherapy sensitivity (8).In an ovarian cancer exosome model, knockdown of TGFBI in TAMs reverses M2 polarization induced by tumor exosomes (57).Inhibiting cathepsin D relieves its suppression of TGFBI, thereby blocking M2 polarization and reshaping the immunosuppressive microenvironment (59).In pancreatic ductal adenocarcinoma, TGFBI has been incorporated into a prognostic gene signature and holds value as a clinical target (64).Overall, targeting TGFBI helps convert the immunosuppressive microenvironment of “cold tumors” into the immune-activated state of “hot tumors,” thereby enhancing the efficacy of immunotherapy and improving patient prognosis.

### 4.2 Inhibitory effect of TGFBI on T cell function and infiltration

TGFBI is a secreted extracellular matrix protein that suppresses T cell function and infiltration in the tumor microenvironment through multiple mechanisms, thereby helping tumors evade immune attack. One major mechanism is the physical barrier formed by TGFBI-rich ECM, which obstructs the entry of cytotoxic T lymphocytes (CTLs) into the tumor parenchyma. Spatial transcriptomic analysis of colorectal cancer shows that TGFBI, together with other ECM regulators, forms an immune exclusion signature (IEX). This signature is associated with chromosomal instability-driven tumor progression and is accompanied by reduced infiltration of cytotoxic immune cells, forming an immune-excluded microenvironment (15).In ovarian cancer, cancer-associated mesenchymal stem cells (CA-MSCs) drive the exclusion of CD8+ T cells from tumor tissue; recruited myeloid cells such as macrophages highly express TGFBI, further creating an immunosuppressive microenvironment and limiting T cell infiltration (61).This physical barrier is a key link in TGFBI-mediated immunosuppression, preventing effector T cells from reaching and killing tumor cells.

In addition to physical barriers, TGFBI can also directly or indirectly inhibit T cell activation and function. Studies have shown that TGFBI is a novel secreted immune checkpoint molecule that directly negatively regulates tumor-infiltrating T cells. Neutralizing TGFBI *in vitro* significantly enhances the activation and cytotoxic capacity of CD4+ and CD8+ T cells from cancer patients. T cells themselves can also express TGFBI, forming an autocrine immunosuppressive loop that inhibits their differentiation into functional effector cells with tissue-homing capacity. In organoid models, blocking TGFBI restores T cell proliferation and tumor-killing ability, confirming its direct inhibitory effect on T cells (12).Meanwhile, TGFBI can also regulate immunosuppression through indirect pathways: in clear cell renal cell carcinoma, TGFBI secreted by tumor epithelial cells upregulates the chemokine CCL22, recruiting FOXP3+ regulatory T cells (Tregs) to form an immunosuppressive microenvironment; neutralizing CCL22 reverses the chemotactic effect on Tregs (11).The interaction of TGFBI with macrophages further amplifies the suppressive effect on T cells. In ovarian cancer, TGFBI secreted by M2-like tumor-associated macrophages inhibits NK cell activity and weakens antitumor immunity (7, 61).Furthermore, TGFBI can indirectly influence the immune checkpoint landscape through pathways such as PD-L1, suppressing effector T cell responses (17). Preclinical studies have strongly supported the anti-tumor effect of TGFBI neutralization *in vivo*. In an orthotopic mouse model of high-grade serous ovarian cancer, treatment with an anti-TGFBI neutralizing antibody reduced tumor burden, increased intratumoral monocyte infiltration, and activated specific T cell subsets, thereby reshaping an immune-permissive microenvironment (7). In an *in vivo* model of CA-MSC-mediated therapy resistance, targeting the Hedgehog pathway to reduce TGFBI-positive myeloid cells restored sensitivity to anti-PD-L1 therapy (61) In colorectal cancer organoids, blocking TGFBI directly enhances T cell cytotoxicity, providing a rationale for the combination of TGFBI-targeting agents with immune checkpoint inhibitors (ICIs) (10). Disrupting TGFBI-mediated physical and biochemical barriers enhances the depth of T cell infiltration and their functionality, overcoming the therapeutic bottlenecks of existing ICIs. Therefore, neutralizing TGFBI or blocking its signaling axis holds promise for converting immune-excluded “cold tumors” into T cell-sensitive “hot tumors,” thereby improving the efficacy of immunotherapy.

### 4.3 Regulation of myeloid-derived suppressor cells and neutrophils by TGFBI

TGFBI plays an important role in the tumor immune microenvironment, particularly affecting myeloid-derived suppressor cells (MDSCs) and neutrophils. In glioblastoma (GBM), a novel interferon-γ-related gene signature that includes TGFBI can be used to define an immune-inflamed phenotype (63).This signature is associated with increased intratumoral infiltration of neutrophils and macrophages, suggesting that TGFBI may be involved in the recruitment or activation of these myeloid cell types (63).Although the existing literature does not detail the specific mechanisms by which TGFBI recruits MDSCs, the association of interferon-γ-related gene signature with a robust innate immune response and multiple immunosuppressive mechanisms suggests that TGFBI may participate in constructing the immunosuppressive microenvironment through a complex network and influence the function of MDSCs (63).In epithelial ovarian cancer, proteomic analysis of platinum-resistant cells reveals that TGFBI is upregulated together with multiple regulators of epithelial-mesenchymal transition (EMT) (65).Bioinformatics analysis of public databases indicates that the expression level of TGFBI in ovarian cancer is positively correlated with the infiltration levels of various immune cells in the tumor microenvironment (65), suggesting that TGFBI may participate in shaping the myeloid cell composition and promoting the accumulation of tumor-associated neutrophils (TANs).

**Figure 3:**
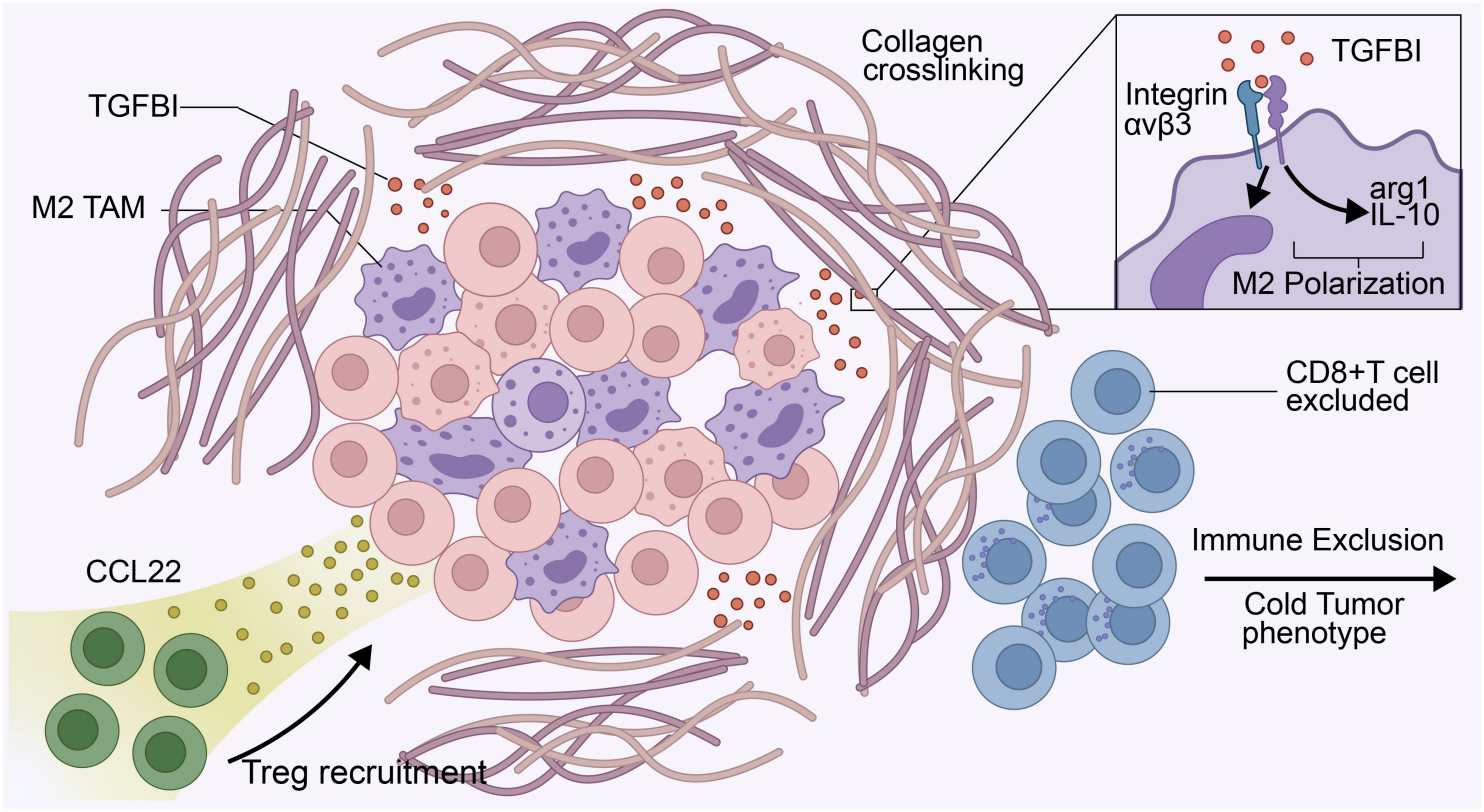
TGFBI remodels the tumor immune microenvironment – immune exclusion and immunosuppression. In the tumor stroma, TGFBI serves as a key molecule that regulates the crosstalk between tumor cells and stromal cells. It induces the polarization of tumor-associated macrophages toward the pro-tumorigenic M2 phenotype, leading to the release of immunosuppressive factors. Concurrently, TGFBI enhances collagen fiber remodeling, increasing extracellular matrix (ECM) stiffness and forming a dense physical barrier that impedes the infiltration of cytotoxic T lymphocytes (CTLs) into the tumor parenchyma, thereby generating a classical “immune-excluded” microenvironment. Furthermore, TGFBI promotes the recruitment of regulatory T cells (Tregs) by inducing CCL22 expression, further reinforcing local immunosuppression.

No studies to date have clearly reported that TGFBI directly induces the polarization of TANs toward the pro-tumor N2 type. However, the association of TGFBI with EMT and neutrophil infiltration provides a theoretical basis for this. EMT is often accompanied by increased tumor invasiveness and the formation of an immunosuppressive microenvironment, which includes N2-type neutrophils that promote angiogenesis and metastasis. TGFBI secreted by TAMs and tumor cells also suppresses NK cell activity and promotes the accumulation of tumor-associated neutrophils (TANs), thereby broadening the immunosuppressive network beyond T cells and macrophages. Therefore, TGFBI may participate in the recruitment and functional polarization of myeloid cells such as MDSCs and TANs by regulating EMT and immune cell infiltration, thereby driving tumor progression and immunosuppression (63, 65). (**Figure 3**).

## 5 Interaction network of TGFBI-related signaling pathways

### 5.1 Integrin-mediated core signaling pathways

In macrophages within the tumor microenvironment, TGFBI upregulates PDGF-B expression via the integrin αvβ3-AKT-ERK pathway, promoting the proliferation and differentiation of macrophages toward a pro-fibrotic phenotype (14).The above evidence indicates that αvβ3/αvβ5 are the core receptors mediating TGFBI’s pro-survival and pro-proliferative signals. In addition to αvβ3/αvβ5, integrin α3β1 is also an important receptor for TGFBI, regulating cell motility and cytoskeletal remodeling. Binding of TGFBI to α3β1 activates Rho GTPase family proteins such as RhoA and Rac1, regulating actin polymerization and cytoskeletal rearrangement, thereby driving cell migration and invasion. Transcriptomic analysis of corneal epithelial cells following TGFBI knockdown shows significant enrichment of cell adhesion molecule and integrin-related signaling, suggesting a compensatory or regulatory role of integrin pathways in cell adhesion (66).Proteomic studies of the developing otic capsule have confirmed that TGFBI is an extracellular protein interacting with integrins and plays a conserved role in cell migration and adhesion-mediated morphogenesis (67).Pan-cancer analysis indicates the important roles of integrins such as ITGB1 and their interacting protein TGFBI in mediating adhesion and signaling during tumor progression and therapy resistance (68).In cholangiocarcinoma, soluble TGFBI exacerbates malignant tumor progression via the integrin β1 (ITGB1)-dependent PPARγ pathway (69).The above studies indicate that binding of TGFBI to integrins such as α3β1 initiates intracellular signals, induces cytoskeletal rearrangement, enhances migratory capacity, and promotes tumor metastasis.

### 5.2 Crosstalk with the TGF-β signaling pathway

TGFBI is a classic downstream target gene of the TGF-β signaling pathway, and its expression is primarily regulated by the canonical TGF-β/Smad pathway, a regulatory relationship that has been validated in various physiological and pathological processes. In cardiac fibroblasts, TGFBI acts as a hub gene in a ceRNA regulatory network, and its expression is closely associated with the TGF-β1 pathway and myocardial fibrosis (70).In a triple-negative breast cancer model, TGF-β1 significantly upregulates TGFBI expression, and the ALK5 inhibitor SB431542 effectively blocks this effect, directly confirming that the transcriptional expression of TGFBI is regulated by TGF-β signaling (71).In the progression of nonalcoholic steatohepatitis-associated liver fibrosis, TGFB1 and TGFBI show concurrent high expression (72); during bovine fetal ovarian development, their expression decreases coordinately, suggesting a synergistic regulatory mechanism (73).In type 2 granular corneal dystrophy, TGF-β1 induces TGFBI protein accumulation in corneal fibroblasts through Smad2/3 phosphorylation (74); in trabecular meshwork cells of the eye, TGF-β2 similarly upregulates TGFBI expression (75).The above studies indicate that TGFBI is a key effector molecule mediating the regulation of fibrosis and extracellular matrix remodeling by TGF-β signaling.

TGFBI is not merely a passive recipient of regulation but can also reciprocally regulate TGF-β signaling and its downstream effects, forming complex feedback loops. The core mechanism involves influencing non-Smad pathways such as MAPK via integrin signaling. In urothelial carcinoma, TGF-β1 upregulates TGFBI expression, which in turn promotes ERK1/2 and p38 phosphorylation, forming a TGFBI-MAPK axis involved in regulating cell proliferation and apoptosis (76).In a cardiac pressure overload model, fibroblast-secreted TGFBI activates macrophages in a paracrine manner, a process negatively regulated by inhibitory Smad7, constituting a TGF-β-driven pro-fibrotic feedforward loop (77).In tumor tissues, TGF-β-induced lncRNA MIR100HG promotes TGF-β1 autocrine signaling, and its expression level is significantly correlated with both TGFB1 and TGFBI, forming a positive feedback loop that amplifies TGF-β signal output (78).In glaucoma, TGF-β2 and autotaxin (ATX) exhibit cross-regulatory interactions, jointly upregulating the expression of TGFBI and TGF-β isoforms, thereby activating the Smad pathway and fibrotic markers (75).

In summary, there is a bidirectional and dynamic regulatory relationship between TGFBI and TGF-β signaling: TGFBI is transcriptionally regulated by the canonical Smad pathway and can in turn modulate TGF-β signaling strength through non-canonical pathways such as integrin-MAPK; together, they participate in the regulation of cell fate during development, fibrosis, and tumorigenesis and progression.

## 6 Strategies and challenges of targeting TGFBI as a therapeutic target in tumors

### 6.1 Monoclonal antibodies and peptide inhibitors targeting TGFBI

Both monoclonal antibodies and peptide inhibitors targeting TGFBI hold good application prospects, as they can specifically block extracellular matrix-driven pro-tumor, pro-metastatic, immune evasion, and pro-fibrotic signaling, providing a theoretical basis for novel therapies for various diseases. Neutralizing monoclonal antibodies (mAbs) targeting specific functional domains of TGFBI represent a potential therapeutic strategy to block its pro-tumor functions, with the core goal of inhibiting the binding of TGFBI to integrin receptors. Preclinical studies have provided experimental validation: in high-grade serous ovarian cancer (HGSOC), TGFBI secreted by tumor-associated macrophages (TAMs) enters the ascites and promotes cancer cell migration and metastasis, while TGFBI neutralizing antibodies can partially inhibit the pro-migratory capacity of TAM-conditioned medium *in vitro (*79);In an orthotopic HGSOC mouse model, anti-TGFBI antibody reduces intraperitoneal tumor volume and reshapes the tumor immune microenvironment by increasing tumor-infiltrating monocytes and activating β3-expressing unconventional T cells (7), combining direct tumor inhibition with reversal of immunosuppression. In clear cell renal cell carcinoma (ccRCC), tumor cell-secreted TGFBI recruits regulatory T cells (Tregs) by upregulating CCL22, promoting immune evasion (11). Although direct antibody experiments have not been reported, the central role of TGFBI in immunosuppression provides a theoretical basis for its neutralization therapy. In liver fibrosis, non-parenchymal cell-derived TGFBI activates hepatic stellate cells and induces macrophage differentiation toward a pro-fibrotic phenotype through integrin αvβ3-mediated signaling (14); theoretically, TGFBI neutralizing antibodies could block this pathogenic cell-cell interaction and pro-fibrotic positive feedback loop. Furthermore, as a secreted immune checkpoint (sIC), TGFBI is widely expressed in the tumor microenvironment and inhibits T cell activation through an autocrine loop; neutralizing TGFBI *in vitro* enhances the activation and differentiation of CD4+ and CD8+ T cells from cancer patients. In colorectal cancer organoid models, blocking TGFBI promotes cytotoxic T cell proliferation and tumor killing (12), making it a target with both direct tumor-inhibitory and immunotherapeutic potential.

In addition to antibody strategies, RGD-containing cyclic peptides or peptide analogs can inhibit the interaction between TGFBI and integrin receptors through competitive binding. The RGD motif is a key sequence for the binding of TGFBI to integrins such as αvβ3 and αvβ5; synthetic peptides can act as decoy ligands to occupy integrin binding sites, thereby blocking the effects of endogenous TGFBI. In cholangiocarcinoma, soluble TGFBI exacerbates malignant progression via the integrin β1 (ITGB1)-dependent PPARγ pathway (69); in pancreatic cancer, TGFBI promotes perineural invasion and epithelial-mesenchymal transition (EMT) through the PI3K/AKT pathway (80). RGD-mimetic peptides could block these pro-tumor signaling cascades at the source. Non-cancerous pathological studies further support this strategy: in liver fibrosis, TGFBI transmits pro-fibrotic signals through integrin αvβ3 on the surface of hepatic stellate cells and macrophages (14); in atherosclerosis, TGFBI is a novel circulating ligand for the scavenger receptors Stabilin-1/2 (Stab1/2) (81), suggesting that peptide inhibitors targeting the FAS1 domain of TGFBI could be designed to block its binding to receptors such as Stab1/2.In corneal dystrophy, mutations in the functional domains of TGFBI lead to protein accumulation and disease (22, 82); shRNA silencing of TGFBI ameliorates the condition (66), while peptide inhibitors could achieve a similar functional knockdown effect by inhibiting the activity of secreted TGFBI without affecting its expression. Both monoclonal antibodies and peptide inhibitors targeting TGFBI hold good application prospects, as they can specifically block extracellular matrix-driven pro-tumor, pro-metastatic, immune evasion, and pro-fibrotic signaling, providing a theoretical basis for novel therapies for various diseases.

### 6.2 Combination therapy strategies

#### 6.2.1 Combination of TGFBI inhibitor with chemotherapy

Combination of TGFBI inhibitors with conventional chemotherapeutic agents may overcome drug resistance by disrupting the extracellular matrix barrier that impedes drug penetration. In GBM, TGFBI is a direct target gene of STAT3, with elevated expression in senescent cells, thereby promoting glycolysis and mediating temozolomide (TMZ) resistance. Knockdown of TGFBI using shRNA inhibits cellular senescence, glycolysis, and TMZ resistance *in vitro*, indicating that targeting the STAT3-TGFBI axis can increase chemotherapy sensitivity—this has been experimentally validated in cell line models (83).In urothelial carcinoma, the synergistic antitumor effect of 5-aza-2’-deoxycytidine (DAC) and cisplatin (CDDP) is achieved through inhibition of the TGFBI-MAPK signaling pathway (76); inhibiting TGFBI enhances the efficacy of platinum-based drugs. In ovarian cancer, TGFBI expression is upregulated in drug-resistant cell lines and is closely associated with resistance to chemotherapies such as topotecan and cisplatin (84, 85).Mechanistically, TGFBI mediates drug resistance by activating the PI3K/Akt pathway, inhibiting apoptosis and promoting DNA repair (42).In colorectal cancer, high expression of TGFBI is associated with greater sensitivity to 5-fluorouracil (5-FU) chemotherapy and better patient prognosis, and may serve as a predictive biomarker for chemotherapy efficacy (86).Piperine can downregulate molecules such as TGFBI and enhance the toxicity of paclitaxel and topotecan in drug-resistant ovarian cancer cells (87), further supporting the feasibility of combining TGFBI modulation with chemotherapy. In gastric cancer, bioinformatic analysis suggests that TGFBI, along with COL4A1 and PXDN, may serve as key markers, and inhibiting their expression is expected to enhance chemotherapy sensitivity (88).In breast cancer polyploid cells, which typically exhibit drug resistance, TGFBI overexpression paradoxically increases paclitaxel sensitivity, indicating that its effect is context-dependent, but inhibiting TGFBI is also effective against certain resistant phenotypes (89).Overall, TGFBI inhibitors can disrupt the protective tumor microenvironment and cell survival pathways, weaken the stromal barrier and intrinsic resistance mechanisms, thereby enhancing the efficacy of chemotherapy in various cancers.

#### 6.2.2 Combination of TGFBI inhibitor with immune checkpoint inhibitors

Combination of TGFBI inhibitors with immune checkpoint inhibitors (ICIs) can help reverse the immunosuppressive tumor microenvironment and improve the efficacy of immunotherapy. TGFBI, primarily secreted by tumor-associated macrophages (TAMs) and cancer-associated mesenchymal stem cells (CA-MSCs), is a key molecule driving immune exclusion and immunosuppression. In glioblastoma, a higher score for the cellular senescence-related gene signature (CSRGS), in which TGFBI is a core component, is associated with worse patient prognosis but also with higher levels of tumor immune infiltration and greater sensitivity to immune checkpoint blockade therapy (83).This suggests that after blocking the immunosuppressive effect of TGFBI, such tumors may respond more effectively to ICI therapy. In ovarian cancer, factors secreted by CA-MSCs lead to CD8+ T cell exclusion and reduce the efficacy of anti-PD-L1 therapy; CA-MSCs and the immunosuppressive myeloid cells they recruit highly express TGFBI, which in turn suppresses NK cell activity (61). Inhibiting Hedgehog signaling in CA-MSCs downregulates TGFBI and restores sensitivity to immunotherapy, directly demonstrating that TGFBI is involved in mediating immunotherapy resistance. In pancreatic cancer, single-cell sequencing shows that TGFBI is secreted by TAMs, and its expression is associated with tumor growth and immunosuppression (8); knockout of TGFBI in macrophages inhibits M2 polarization, enhances antitumor immunity, and increases chemotherapy sensitivity. Glioma research shows that the nicotinamide and tryptophan metabolism pathways, which are associated with poor prognosis and high immune infiltration, are centered on TGFBI as a core gene; a risk model incorporating TGFBI can predict the efficacy of immunotherapy. *In vitro* and *in vivo* experiments confirm that high TGFBI expression promotes glioma cell migration, invasion, and EMT (90).In head and neck squamous cell carcinoma, TGFBI has been identified as a potential tumor-shared antigen, highly expressed in tumor cells and often accompanied by HLA class I molecule expression, making it a target for tumor vaccines or T cell therapy that can act synergistically with ICIs (91).In esophageal adenocarcinoma, hub genes such as TGFBI are progressively upregulated with disease progression and may serve as biomarkers for predicting the efficacy of immune checkpoint blockade (ICB) therapy (92).In ovarian cancer, platinum-resistant cells exhibit EMT and metabolic reprogramming, with significantly upregulated TGFBI expression, which correlates with the infiltration of CD8+ T cells, macrophages, neutrophils, and dendritic cells in the tumor microenvironment, indicating that TGFBI is involved in both drug resistance regulation and immune microenvironment remodeling (65).

TGFBI is a core molecule mediating tumor immunosuppression. TGFBI inhibitors can reverse T cell exclusion, reduce the activity of myeloid suppressor cells, and enhance the efficacy of PD-1/PD-L1 or CTLA-4 inhibitors. The combination of the two is expected to become an important frontier strategy in tumor immunotherapy.

#### 6.2.3 Combination of TGFBI inhibitor with anti-angiogenic drugs

Combination of TGFBI inhibitors with anti-angiogenic agents can simultaneously target angiogenesis and the stromal supportive microenvironment through complementary pathways, disrupting tumor blood vessel formation. As an extracellular matrix protein, TGFBI exerts multiple roles in regulating the tumor microenvironment.

In renal cell carcinoma bone metastasis (RCCBM), tumor cell-secreted BIGH3/TGFBI disrupts bone homeostasis by inhibiting osteoblast differentiation and inducing osteocyte apoptosis, thereby driving osteolytic progression (93).Although this study focuses on bone metastasis, it suggests that TGFBI may affect vascular microenvironment stability in a similar manner. The TGFBI-mediated effects can be reversed by bone anabolic agents but not by lysosomal inhibitors, providing specific pathway targets for combination therapy (93).An engineered 3D hydrogel model of glioblastoma shows that TGFBI secreted by M2-polarized macrophages acts as a pro-tumor factor, promoting the formation of the invasive mesenchymal subtype (58).Tumor invasion and angiogenesis are often interrelated, and targeting macrophage-derived TGFBI may achieve vascular normalization, thereby improving the delivery efficiency and efficacy of anti-angiogenic drugs. In ovarian cancer, TGFBI expression is upregulated in drug-resistant cell lines and is associated with chemotherapy resistance, mediated in part through pathways such as PI3K/Akt that are also involved in angiogenesis (42, 84).Therefore, combining TGFBI inhibitors with anti-angiogenic drugs such as bevacizumab can simultaneously target stromal support signals and vascular supply. A meta-analysis in gastric cancer indicates that TGFBI, along with COL4A1 and PXDN, are key molecules associated with drug resistance, with their interactions enriched in extracellular matrix-related pathways (88).COL4A1 is a key component of the basement membrane for maintaining vascular integrity; combining targeting of TGFBI with anti-angiogenic agents that affect collagen deposition or VEGF signaling may produce synergistic effects. In breast cancer polyploid cells, TGFBI influences paclitaxel sensitivity and participates in polyploid formation, processes that can alter tumor vascular structure and hemodynamics (89).Studies on orthopoxvirus infection show that lactoferrin restores TGFBI expression and activates the MAPK/ERK and JAK2/STAT3 pathways, indicating that TGFBI is involved in regulating a broad signaling network, including endothelial cell-related responses (94).Although this study is not focused on tumors, it confirms that TGFBI can regulate key pathways potentially related to angiogenesis. Overall, combined inhibition of TGFBI and angiogenic pathways can disrupt the stability of the tumor vascular network, block nutrient supply, and improve the penetration efficiency and efficacy of other therapeutic agents, achieving a comprehensive anti-tumor effect (**Table 2**).

**Table 2:** Preclinical evidence for TGFBI-targeted therapeutic strategies.

Therapeutic strategy	Cancer model	Modality	Key outcome	Evidence level	Translational limitations	Reference
Anti-TGFBI mAb	HGSOC (orthotopic mouse)	In vivo	Reduced tumor burden, activated β3+ T cells	Experimental validation	Long-term safety unknown; systemic delivery	(5)
Anti-TGFBI mAb	HGSOC (in vitro)	In vitro	Inhibited TAM-conditioned medium-induced migration	Experimental validation	Lacks in vivo confirmation in this specific context	(77)
Anti-TGFBI mAb	Colorectal cancer organoids	Ex vivo	Enhanced CTL proliferation and tumor killing	Experimental validation	Organoid model limitations; lacks in vivo data	(10)
Anti-TGFBI mAb	ccRCC	N/A	Predicted to block Treg recruitment	Hypothetical	No direct experimental testing	(9)
TGFBI mAb + anti-PD-L1	Ovarian cancer (CA-MSC model)	In vivo	Hedgehog inhibition→↓TGFBI→restored anti-PD-L1 sensitivity	Experimental validation	Indirect TGFBI targeting; Hedgehog pathway specificity	(59)
TGFBI KO + chemo	Pancreatic cancer (GEMM)	In vivo	Inhibited M2 polarization, enhanced chemo-sensitivity	Experimental validation	Genetic model; translatability to pharmacologic inhibition unclear	(6)
TGFBI shRNA + TMZ	GBM (cell lines)	In vitro	Inhibited glycolysis and TMZ resistance	Experimental validation	shRNA not clinically applicable; lacks in vivo data	(81)
TGFBI modulation + chemo	Ovarian cancer (drug-resistant cells)	In vitro	Piperine↓TGFBI enhanced paclitaxel/topotecan	Experimental validation	Piperine has multiple targets; specificity unclear	(85)
RGD-mimetic peptides	Various solid tumors	N/A	Predicted to block TGFBI-integrin binding	Hypothetical	No experimental testing in cancer models; specificity concerns	(67, 78)

### 6.3 Challenges and translational prospects

The dual functions of TGFBI and its dependence on the tissue microenvironment represent major challenges for clinical translation. To avoid treatment inefficacy or adverse effects, precise stratification of patients based on reliable biomarkers is necessary.

Biomarker-based patient stratification is critical. In non-small cell lung cancer, TGFBI is a key gene mediating radioresistance and poor prognosis, and is associated with immune infiltration patterns such as those of naive B cells and M0 macrophages (95).In gastric cancer, high stromal TGFBI expression in the presence of lipopolysaccharide indicates a worse prognosis and may be associated with resistance to nivolumab (96).These results indicate that a combination of factors, including TGFBI expression level, cellular origin (tumor cells, CAFs, TAMs, etc.), and immune microenvironment features, is needed to screen the populations most likely to benefit from TGFBI-targeted therapy. Systemic inhibition of TGFBI may interfere with its normal physiological functions in tissues such as the cornea and bone, leading to adverse effects including corneal dystrophy and bone metabolic abnormalities. Therefore, developing locally delivered or conditionally activated inhibitors represents an important translational strategy. TGFBI is the second most abundant protein in the corneal stroma, and its mutations can lead to various autosomal dominant corneal dystrophies, including lattice and granular types (18).In bone tissue, TGFBI (BIGH3) is involved in bone formation and homeostasis maintenance; TGFBI secreted by renal cell carcinoma cells can drive osteolytic bone metastasis by inducing osteocyte apoptosis and disrupting bone remodeling (93). Systemic targeting may induce corneal pathology or exacerbate bone metabolic abnormalities.

Local delivery strategies are currently being explored in corneal diseases. In a mouse model of granular corneal dystrophy, topical administration using hybrid lipid nanoparticles encapsulating siRNA effectively silenced mutant TGFBI (R124H) (97).CRISPR adenine base editing (ABE) can efficiently correct common TGFBI mutations (R124C, R555W) *in situ* in human corneal epithelial cells with extremely low off-target effects, laying the foundation for local gene therapy (98).In cancer therapy, the use of nanocarriers or engineered delivery systems to selectively release TGFBI inhibitors within the tumor microenvironment can reduce systemic exposure. Studies have also found that TGFBI is enriched in a class of extracellular nanoparticles termed “supermeres,” which have higher *in vivo* uptake efficiency than exosomes and possess unique functions, potentially providing a novel natural carrier for targeted drug delivery or guiding local therapy (99).These strategies all aim to achieve precise targeting and reduce off-target toxicity. Compared with directly targeting the full-length TGFBI protein, selectively blocking its downstream tumor-specific pro-tumor signaling pathways represents another direction to improve therapeutic selectivity and efficacy. TGFBI binds to different integrins to activate multiple intracellular signals, primarily mediating invasion, metastasis, immunosuppression, and therapy resistance in tumors.

**Figure 4:**
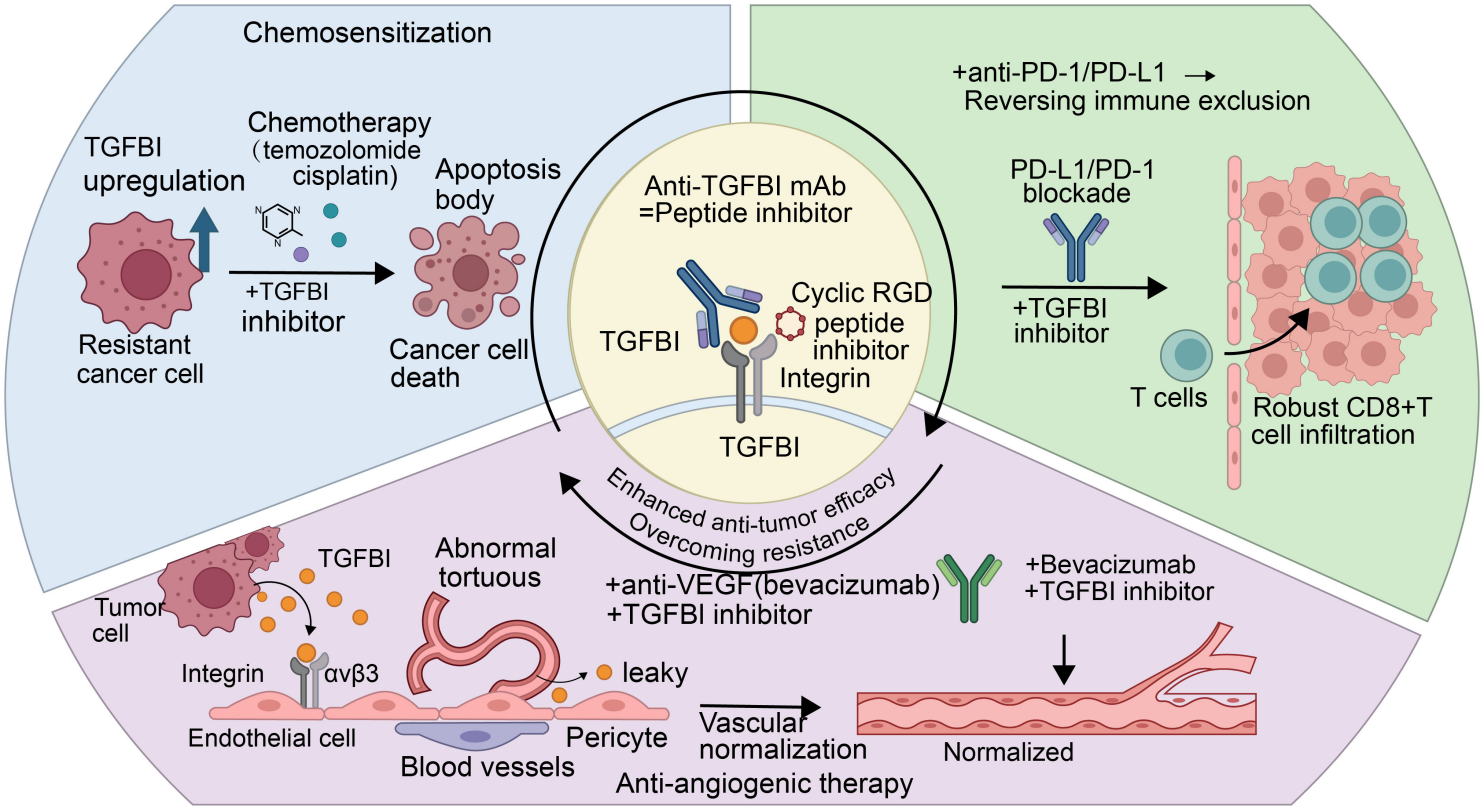
Circular schematic of integrated therapeutic strategies targeting TGFBI in cancer. Circular summary of TGFBI−targeted combination therapies. The central circle shows anti−TGFBI antibodies and peptide inhibitors blocking integrin αvβ3. Three surrounding sectors depict: (1) chemosensitization: TGFBI inhibitor overcomes drug resistance and induces apoptosis; (2) immune checkpoint inhibition: co−targeting TGFBI and PD−1/PD−L1 reverses CD8^+^ T−cell exclusion; (3) anti−angiogenic therapy: combining TGFBI inhibitor with bevacizumab normalizes tumor vasculature. The overall synergy leads to enhanced anti−tumor efficacy and resistance reversal.

These studies suggest that blocking specific receptor binding or downstream effector molecules could selectively inhibit the pro-tumor effects of TGFBI while preserving its physiological functions. Therefore, selectively targeting these regulatory nodes or downstream pathways in combination with immune checkpoint inhibitors, chemotherapy, or other regimens may effectively address the challenges posed by the dual functionality of TGFBI, achieving safer and more efficient cancer therapy. (**Figure 4**).

## 7 Conclusion

TGFBI is a key extracellular matrix protein whose function is highly dependent on the microenvironment, and it plays multiple roles in tumor biology, which presents both challenges and opportunities. The understanding of this protein reflects a shift in cancer research from the simplistic view that a molecule is either pro-tumor or anti-tumor to a more complex perspective where the microenvironment determines its dynamic, dual functions. To realize its clinical value, integrated research from multiple aspects is needed. Single-cell RNA sequencing (scRNA-seq) and spatial transcriptomics have emerged as powerful tools for dissecting TGFBI function at cellular resolution. scRNA-seq enables precise localization of TGFBI expression in specific cell subsets within the tumor microenvironment, such as partial-EMT tumor cells and tumor-associated macrophages. Spatial transcriptomics complements this by placing TGFBI expression in the context of specific anatomical microenvironments, including the tumor invasive front, perivascular niches, and immune-excluded zones, revealing how TGFBI function is spatially regulated. Integration of these advanced technologies with functional studies will be essential for translating TGFBI-based biomarkers and therapeutic strategies into clinical practice.

Early work mainly observed the aberrant expression of TGFBI, whereas current research has advanced into molecular mechanisms and therapeutic exploration. The dual faces of its function depend on the cancer type, stage, and microenvironment. The core mechanism involves binding to different integrin receptors, activating downstream pathways such as FAK and PI3K/AKT, and regulating tumor proliferation, EMT, and metastasis; it can also influence immune components such as macrophages and T cells, creating an immunosuppressive microenvironment. The integrin expression profile, stromal interactions, and epigenetic modifications collectively determine whether it ultimately promotes or inhibits tumors. Research should avoid overgeneralization and pay attention to tumor heterogeneity and the dynamic changes of the microenvironment. In the era of immunotherapy, TGFBI represented an important target for combination therapy. Targeting it can relieve immunosuppression, and combining it with immune checkpoint inhibitors, chemotherapy, or radiotherapy can produce synergistic effects and overcome resistance. However, it is also necessary to balance its normal physiological functions in tissue homeostasis and injury repair to prevent adverse effects. Future efforts require the development of tumor-specific delivery systems and the promotion of multidisciplinary translational collaboration. Going forward, research should focus on the functional heterogeneity of TGFBI, utilizing technologies such as single-cell sequencing and spatial transcriptomics to dissect its specific roles in different tumors and to screen for precise biomarkers that guide individualized therapy. Concurrently, small molecules or antibodies that specifically block TGFBI-integrin interactions should be developed, and through interdisciplinary collaboration, its biological functions should be systematically understood. The complexity of TGFBI reflects the overall complexity of the tumor microenvironment. Following the principles of precision medicine, designing targeting strategies based on individual patient characteristics and combining them with existing therapies holds promise for improving prognosis. As its regulatory mechanisms are progressively elucidated, TGFBI has the potential to become one of the core targets in the next generation of individualized cancer diagnosis and therapy.
